# Does cumulative adverse socioeconomic exposure mediate the association of maternal mental ill-health at birth and adolescent mental ill-health at age 17? An analysis of the Millennium Cohort Study

**DOI:** 10.1136/jech-2022-220248

**Published:** 2023-06-06

**Authors:** Faye Sheldon, Ben Barr, Sophie Wickham

**Affiliations:** Department of Public Health, Policy and Systems, University of Liverpool, The Farr Institute @ the Health eResearch Centre, Liverpool, UK; Department of Public Health, Policy and Systems, University of Liverpool, The Farr Institute @ the Health eResearch Centre, Liverpool L69 3GL, UK

**Keywords:** Social class, maternal health, adolescent, mental health, poverty

## Abstract

**Background:**

Adolescent mental health is a public health priority. Maternal mental ill-health and adverse socioeconomic exposure (ASE) are known risk factors of adolescent mental ill-health. However, little is known about the extent to which cumulative ASE over the life-course mediates the maternal-adolescent mental health association, which this study aims to explore.

**Methods:**

We analysed data from more than 5,000 children across seven waves of the UK Millennium Cohort Study. Adolescent mental ill-health was measured using the Kessler 6 (K6) and Strengths and Difficulties Questionnaire (SDQ) at age 17. The exposure was maternal mental ill-health as measured by the Malaise Inventory at the child’s birth. Mediators were three measures of cumulative ASE defined by maternal employment, housing tenure and household poverty. Confounders measured at 9 months were also adjusted for, these were: maternal age, maternal ethnicity, household poverty, maternal employment, housing tenure, maternal complications during labour and maternal education. Using causal mediation analysis, we assessed the cumulative impact of ASE on the maternal-adolescent mental-ill health relationship between birth and age 17.

**Results:**

The study found a crude association between mothers’ mental health at the child’s birth and mental health of their children at age 17, however when adjusting for confounders this association was reduced and no longer significant. We did not find an association between cumulative exposure to maternal non-employment or unstable housing over the child’s life-course and adolescent mental health, however cumulative poverty was associated with adolescent mental ill-health [K6: 1.15 (1.04,1.26), SDQ: 1.16 (1.05,1.27)]. Including the cumulative ASE measures as mediators reduced the association between maternal and adolescent mental health, but only by a small amount.

**Conclusions:**

We find little evidence of a mediation effect from cumulative ASE measures. Experiencing cumulative poverty between the ages of 3 to 14 was associated with increased risk of adolescent mental ill-health at age 17, suggesting actions alleviating poverty during childhood may reduce adolescent mental health problems.

## Introduction

Adolescent mental ill-health is estimated to affect ∽15% of UK adolescents (1). Consequences of poor mental health in adolescents are far-reaching and span the life-course with evidence suggesting an association with poor mental health in adulthood, job loss, poor physical health, and an increased demand on services including health and social care into adulthood (2). There is strong evidence that factors such as poor maternal health (3), poor social economic status, and living in poverty (4), negatively impact adolescent mental health (5,6).

Although we know that adverse socioeconomic exposure (ASE) is associated with mental health (5,6), little is known about how *cumulative* ASE throughout an adolescent’s life-course may mediate the maternal-adolescent mental health association. There is a social gradient, so that by age 11, 17% children in the lowest income quintile have severe mental ill-health, compared to just 4% in the highest income quintile (7). Understanding the cumulative effect of ASE on mental health could provide evidence for earlier, targeted policy intervention to reduce health inequalities and protect this vulnerable group (8).

There is an urgent need to improve adolescent mental health, which may feasibly be addressed through policy tackling the known causes of mental ill-health (8). Evidence shows that exposure to prolonged adverse experiences in childhood, including living in poverty and parental mental ill-health, results in a 6-fold higher risk of poorer mental health by age 14 (9). Although existing literature demonstrates the association between ASE and maternal mental ill-health, and the association between maternal and adolescent mental ill-health when adjusting for ASE as a confounder, our analysis with cumulative ASE as a mediator helps to indicate whether living in persistent poverty may be causing mental ill health by age 17. Existing literature cover a range of ages that do not focus on the sensitive transition period of age 17, and do not consider the impact that cumulative ASE has throughout adolescence. Furthermore, by focusing on children of mothers who suffer from mental ill-health around the time of their birth, we also provide evidence to offer greater mental health support as part of maternity services, particularly among mothers who are living in poverty. We assess the extent to which measures of *cumulative* ASE mediate the association between maternal mental ill-health at 9 months and adolescent mental ill-health at age 17, as well as the association of cumulative ASE and adolescent mental health, using the UK Millennium Cohort Study (MCS).

## Methods

### Study Design and Population

**Figure 1:**
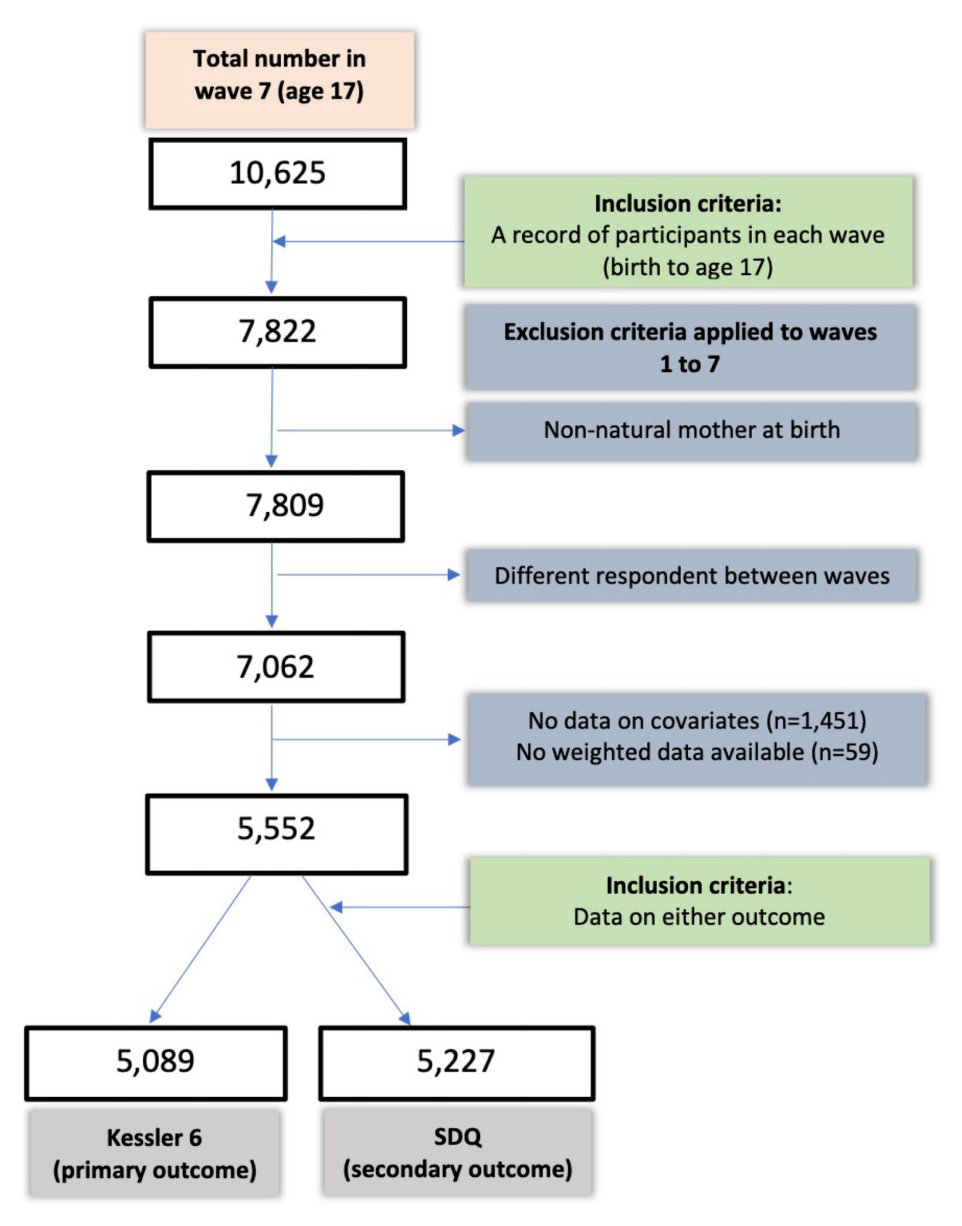
A flow diagram to demonstrate the final sample size after application of inclusion and exclusion criteria, starting with the total number in the 7^th^ data collection wave. Abbreviations: SDQ = Strengths & Difficulties Questionnaire.

The MCS is a nationally representative dataset with a large cohort born in the UK between 2000-2002 (10). Seven waves of data have been collected: 9 months (wave 1), 3 years (wave 2), 5 years (wave 3), 7 years (wave 4), 11 years (wave 5), 14 years (wave 6) and 17 years (wave 7) (11). A clustered stratified sampling design oversampled individuals from ethnic minorities and disadvantaged areas (12,13). We included 5,089 adolescents (48% of sample entering wave 7, see Figure 1) that met the eligibility criteria for the primary outcome (K6) and 5,227 for the secondary outcome (SDQ).

### Measures

#### Exposure (maternal mental-ill health)

Our exposure was the modified Malaise Inventory (MI) as a measure of maternal mental ill-health at MCS birth (9 months) (14). A 9-item questionnaire, it asks about symptoms such as whether respondents feel “tired most of the time”, “miserable or depressed”, or get “easily upset or irritated” (14–16). Respondents answer either yes (1) or no (0), producing a total score of 0-9 (14,15). The 9-item modified MI has moderate discriminant validity for “psychiatric disorder” (17). A score ≥4 was applied to indicate higher risk of maternal depression and/or anxiety (coded as 1 in our analysis) concurring with existing MCS literature (14). Scores <4 were coded as 0.

#### Outcome (adolescent mental ill-health)

We included two measures of adolescent mental ill-health at age 17. The primary outcome was K6, a 6-item questionnaire producing a score of 0-24 (18). It measures self-reported depression and anxiety symptoms experienced within the previous 30 days (19). Severe mental ill-health is considered present among individuals scoring ≥13 (18,20). K6 has good internal consistency (α=0.89) and high classification accuracy (0.92) using this threshold (18,20). In our analysis, ≥13 was coded as 1, and <13 as 0.

The secondary outcome was SDQ, a 25-item questionnaire measuring socioemotional behavioural problems within the last 6 months (21). There are 5 domains assessing emotion, conduct, hyperactivity, relationships with others and prosocial behaviour (22). The total score is out of 40, with ≥17 considered high or very high, and indicative of socioemotional behavioural problems (23,24). Scores ≥17 were coded as 1, and <17 as 0. Both the K6 and SDQ were asked of cohort members themselves for the first time at age 17. Therefore, both measures represent self-reported mental health.

#### Mediators: cumulative ASE

Mediators were chosen *a priori* based on previous literature as the most appropriate markers of ASE (25). Three markers of cumulative ASE were assessed between MCS waves 2-6: maternal employment, housing tenure and household poverty (26). Each mediator was coded as 0 or 1, representing lower or higher ASE respectively. Employment was measured by maternal-reported employment status, i.e. employed (0) or not employed (1) (26). Housing tenure was measured according to whether mothers owned or privately rented their house (0), or lived in more unstable accommodation such as social rental, or squatting (1) (26). Household poverty was defined as income less than 60% median equivalised using the Organisation for Economic Cooperation and Development (OECD) (1), compared to median incomes >60% (0) (19). Scores were summed across waves, giving each mediator a cumulative score out of 5 indicating the number of waves the child was “exposed” to ASE (see supplementary material 1 (S1)).

Our additional analysis included child mental health (measured by proxy-reported SDQ between ages 3-14, scored as above) and child development (measured by Bracken school readiness at age 3) as mediators. These were identified as factors relating to the maternal-adolescent mental ill-health pathway (27).

#### Confounders

**Figure 2:**
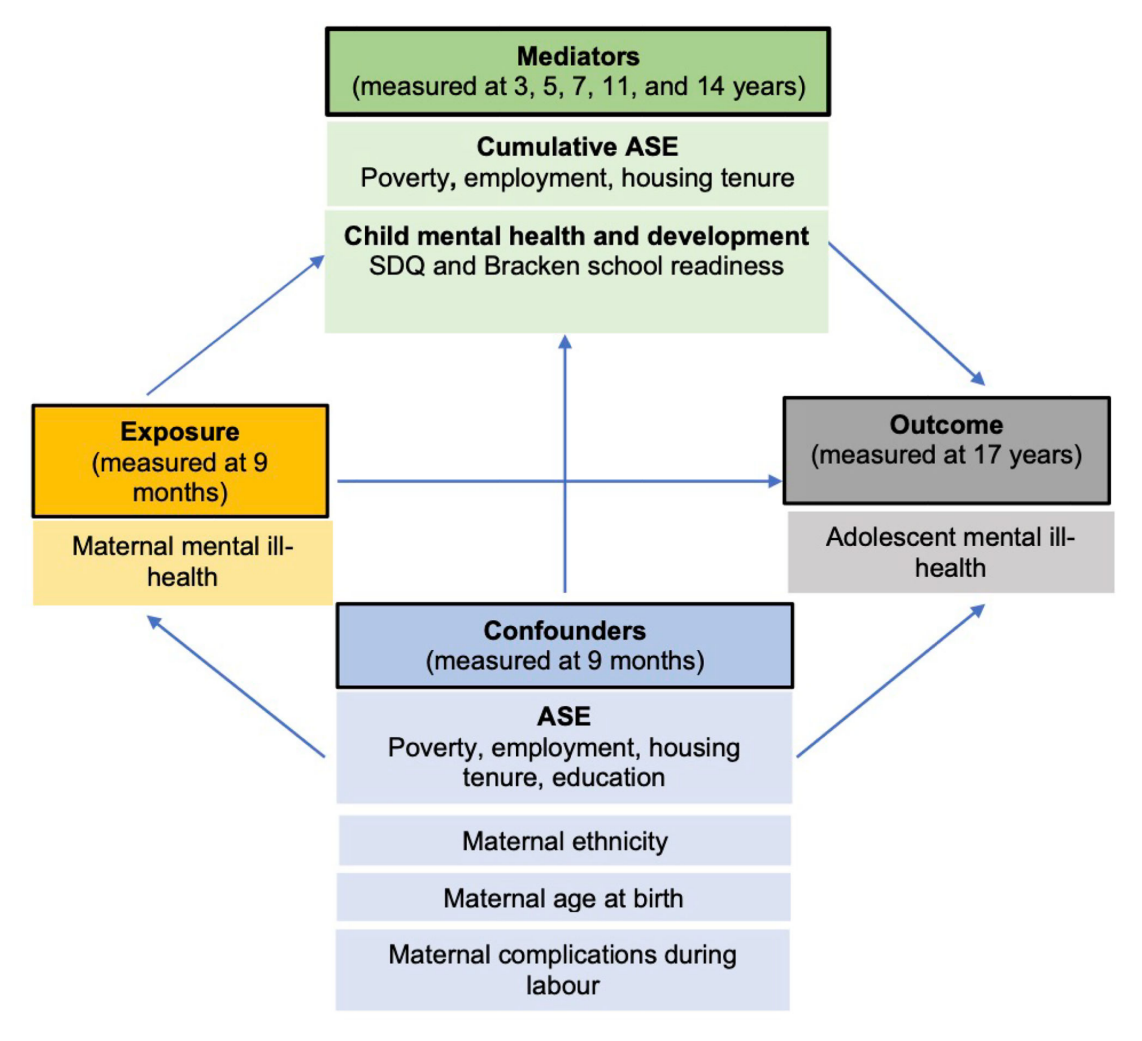
Directed Acyclic Graph showing mediator and confounding variables of the relationship between exposure (maternal mental ill-health at 9 months) and outcome (adolescent mental ill-health at age 17). Abbreviations: ASE = Adverse Socioeconomic Exposure, SDQ = Strengths & Difficulties Questionnaire.

Confounders were identified *a priori* from existing literature (23,28). Measured at 9 months, these were: maternal age, maternal ethnicity, household poverty, maternal employment, housing tenure, maternal complications during labour and maternal education. As per other variables in the analysis, each confounder was coded as 0 (lower ASE) or 1 (higher ASE). Mediators and confounders are depicted in the Directed Acyclic Graph (DAG) (Figure 2).

#### Statistical Analysis

All analyses were carried out using R (version 4.0.2). Mediation analysis was used to determine the extent to which measures of cumulative ASE explain the association between maternal mental ill-health at MCS birth, and adolescent mental ill-health at age 17. A Baron and Kenny mediation approach informed Models 1, 2 and 3, and their interpretation, requiring 4 logistic regressions (29,30): maternal mental ill-health as a predictor of adolescent mental ill-health (path c), maternal mental ill-health as a predictor of cumulative ASE (path a), cumulative ASE as predictors of adolescent mental ill-health (path b) and maternal mental ill-health and cumulative ASE as predictors of adolescent mental ill-health (path a*b). As ASE is potentially both a confounder and mediator of the maternal mental ill-health and adolescent mental ill-health relationship, we attempted to separate these pathways by including ASE at baseline (9 months) and cumulative ASE at 3,5,7,11 and 14 years. This helped to isolate the effect of accumulation of disadvantage after 9 months, and so it is only this effect that could be a mediator, rather than effects of poverty during and before the perinatal period. Outcomes were coded into binary formats for logistic regression analyses. Survey weights were applied to analyses which enabled adjustment for the clustered, stratified sampling design and attrition bias (12,13).

#### Robustness tests

An additional weighted logistic regression analysis with child mental health and development mediators was performed. This was to test if mediation by measures of cumulative ASE, if observed, still held when additionally controlling for mediation by child mental health and development, and whether any observed mediation by cumulative ASE was independent of mediation by child mental health and development. Two sensitivity analyses were performed: weighted linear regression to test the robustness of the binary cut-off scores used in logistic regression models, and unweighted logistic regression to test the robustness of weighted effect size estimates.

## Results

In our sample, 16% (817 individuals, measured by K6) and 17% (871, measured by SDQ) experienced mental ill-health at age 17. 13.3% (109, K6) and 14.0% (122, SDQ) had mothers with mental ill-health at 9 months. A trend of increasing median adolescent mental ill-health at age 17 with increasing maternal mental ill-health score at 9 months was observed (see S2).

Although levels of maternal mental ill-health were reasonably similar across the two groups (13.3% vs. 11.3%, p=0.114), adolescents with mental ill-health were more likely to have mothers with lower or no education, be in poverty, and less likely to live in stable housing. School readiness, maternal age and ethnicity were also similar in their distribution between adolescents with and without mental ill-health (see Table 1). Adolescents with mental ill-health measured by SDQ (see S3) were more likely to have mothers who were younger, of white ethnicity, unemployed and with mental ill-health at MCS birth.

Table 2 shows weighted logistic regression models and further analyses for both primary (K6) and secondary (SDQ) outcomes.

In our unadjusted analysis, there was a relatively large association between maternal and adolescent mental ill-health for the K6: 1.23 (0.94,1.60), and for the SDQ: 1.38 (1.07,1.78), but only reaching statistical significance for SDQ.

Adjusting for confounders considerably reduced effect sizes from 1.23 to 1.15 for K6, and from 1.38 to 1.24 for SDQ. There were significant associations between maternal mental ill-health and each of the mediator variables (see S4), suggesting that poorer maternal mental ill-health may predict higher ASE. The regression assessing mediators as predictors of adolescent mental ill-health (see Table 3) showed that cumulative poverty was the strongest predictor out of the cumulative ASE mediators, and was significant for both K6: 1.15 (1.04,1.26) and SDQ: 1.16 (1.06,1.27). Additionally adjusting for mediation by three measures of cumulative ASE did not markedly change the effect sizes further for either K6 (from 1.15 to 1.11) or SDQ (from 1.24 to 1.19).

Model 2 showed a strong effect size for poverty at 9 months predicting adolescent mental ill-health for both K6: 1.29 (0.99,1.69) and SDQ: 1.28 (0.99,1.65), but neither reached significance. Maternal complications during labour, and housing at 9 months were significant predictors of adolescent mental ill-health when measured by SDQ: 1.21 (1.01,1.45) and 1.45 (1.15,1.83) respectively.

However, when also controlling for the three cumulative ASE mediators in Model 3, *cumulative* poverty became more important than poverty at 9 months, and was the most important cumulative ASE variable, significantly predicting adolescent mental ill-health for both K6: 1.15 (1.04,1.26) and SDQ: 1.16 (1.05,1.27). Maternal complications during labour also remained an important predictor of mental ill-health in Model 3, which although was not significant for K6: 1.14 (0.95,1.37), was significant for SDQ: 1.21 (1.01,1.45). Similar findings were seen in unweighted logistic analyses (see S5).

### Robustness tests

#### Additional analysis: inclusion of child mental health and development mediators

Adding child mental health and development mediators into Model 3 (see S6), substantially reduced effect sizes from Model 2, from 1.15 (0.88,1.50) to 1.05 (0.80,1.39) for K6, and from 1.24 (0.95,1.61) to 0.99 (0.75,1.30) for SDQ. A large attenuation of effect size indicates that child mental health and development are important mediators, explaining most of the association between maternal and adolescent mental ill-health (29,30).

#### Sensitivity analysis: weighted linear regression

Linear regression was performed on untransformed data to prevent loss of information from outcome variable scores of 0. Linear regression found similar results to logistic regression (see S7). The unadjusted maternal-adolescent mental ill-health association was even stronger, being significant for both K6: β 1.06 (0.57,1.55) and SDQ: β 1.20 (0.65,1.76). The largest reduction in effect size occurred when adjusting for confounders, which was almost twice as large with SDQ compared to K6, from β 1.20 (0.65,1.76) to 0.89 (0.34,1.45) and from β 1.06 (0.57,1.55) to 0.92 (0.42,1.42) respectively. As was seen in logistic regression, there was a much smaller reduction in effect size when additionally adjusting for three cumulative ASE variables for both K6: β 0.92 to 0.85 and SDQ: β 0.89 to 0.79. Cumulative poverty had the largest effect size for K6: β 0.24 (0.05,0.42) and SDQ: β 0.40 (0.20,0.61) out of the cumulative ASE variables.

#### Likelihood Ratio Test

A likelihood ratio test demonstrated that Model 3 was the best fit, indicating that adding in cumulative ASE variables are a cause of adolescent mental ill-health (but not necessarily mediators). Model 3 was also the best fit in our additional analysis including child mental health and development mediators.

## Discussion

Using data from a UK-wide large birth cohort study, we found that there is an association between maternal and adolescent mental ill-health, however cumulative ASE (measured by three different measures) does not mediate this relationship. When controlling for high ASE at birth, we showed that cumulative poverty was strongly predictive of adolescent mental ill-health. Maternal complications during labour were also found to predict adolescent mental ill-health. Our additional analysis found that child mental health and development were important mediators in the association between maternal and adolescent mental ill-health.

**Figure 3:**
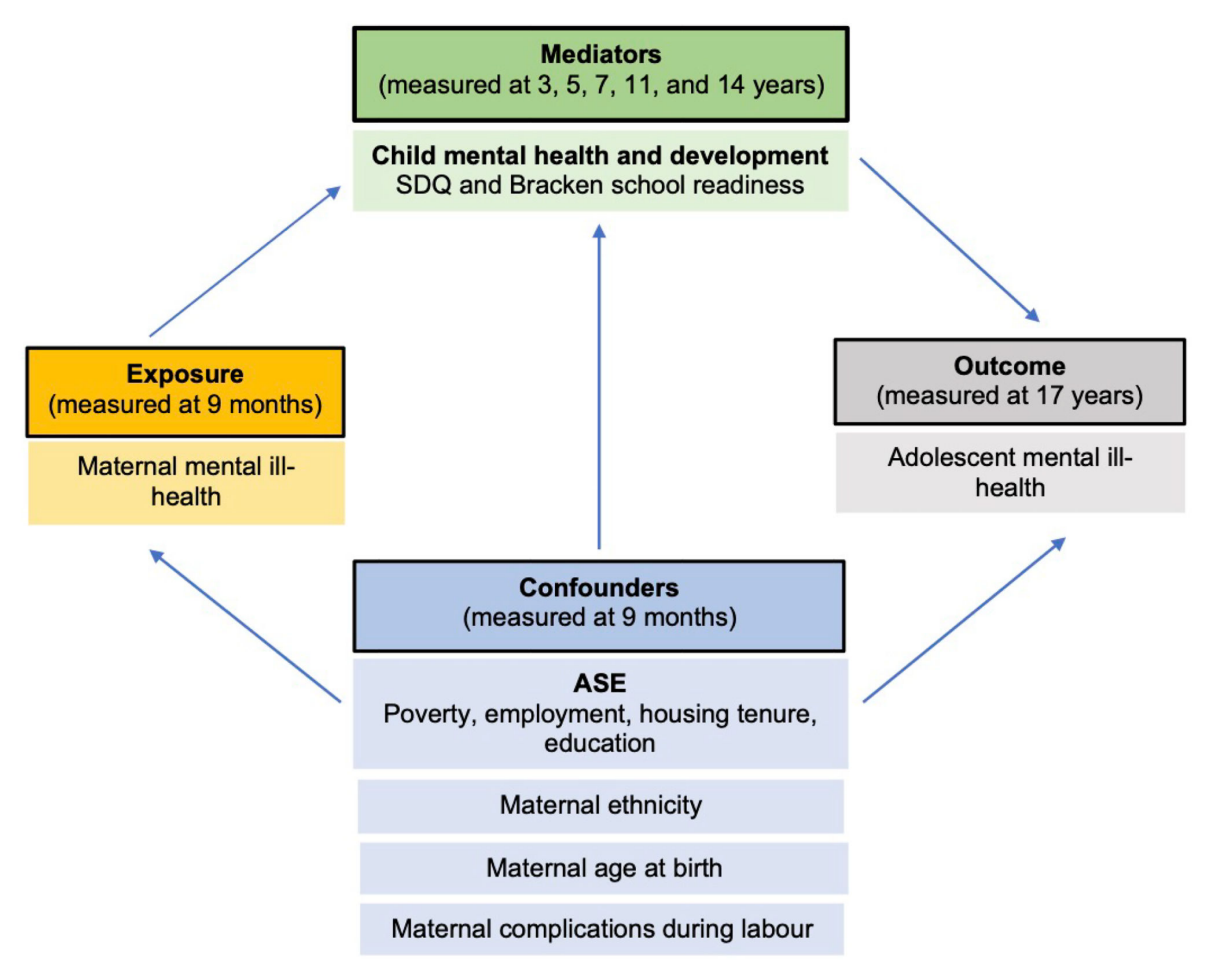
Post-analysis revised Directed Acyclic Graph based on findings from our study. Abbreviations: ASE = adverse socioeconomic exposure, SDQ = Strengths and Difficulties Questionnaire

We know that mental ill-health as measured in our analysis encompasses low mood and socioemotional behavioural problems, through to clinical diagnoses of depression (31). The proposed pathways by our findings are that maternal mental ill-health impacts a child’s mental health and development early in life, leading to poorer adolescent mental health outcomes. Furthermore, when controlling for poverty at birth, being in persistent poverty had a detrimental impact on adolescent mental health by age 17. Considering our results, a revised DAG was produced (Figure 3).

### Findings in the context of current literature

Our results support the role of poverty in causing mental ill-health among adolescents. Our findings concur with Wickham et al., whereby transitioning into poverty was associated with poor mental health up to age 11, and this was attenuated when controlling for maternal mental health (36). Additionally, Lai et al. and Rees showed 3 and 4-fold increases respectively in mental ill-health and emotional and behavioural difficulties among children in persistent poverty compared to no poverty at age 14 (37,38). This risk increased to 6-fold in Adjei et al.’s study when persistent poverty was combined with parental mental ill-health (9).

Current literature shows that by age 14, children experiencing poverty and parental mental ill-health have poorer mental health themselves (9). Our analysis builds upon this, finding that maternal mental health impacts adolescent mental health through child mental health, and that adolescent mental health is directly worsened by increased time in poverty. The role of cumulative poverty directly worsening adolescent mental health is supported by a life-course pathway, whereby risk accumulates and ultimately widens health inequalities (8,39).

Most existing studies test maternal mental health as a mediator of the ASE-adolescent mental health association (4,23,28,36,42,43). Our study corroborates previous studies regarding the role of confounders, such as poverty at 9 months, on maternal and adolescent mental health. Whilst our findings dispute cumulative ASE as a mediator of the maternal-adolescent mental health association, we provide evidence for another pathway through child mental health and development as mediators of the maternal-adolescent mental health association. Existing studies have only addressed part of this pathway.

Our analysis also indicates that maternal complications during labour are an important predictor of adolescent mental ill-health. There is some evidence that mothers who experience labour complications have poorer subsequent mental health (40,41). We also know that there is a direct association between maternal and adolescent mental health. Therefore, it is possible that our findings are occurring through maternal mental ill-health. However, this pathway was outside the scope of our analysis.

Trends in adolescent mental ill-health are worsening following the COVID-19 pandemic and the cost-of-living crisis, with reports of up to 20% of 17-22 year olds experiencing mental ill-health (1). Possible causes of declining mental health in adolescents include prolonged social isolation and reduced social contact during large periods of the pandemic, in addition to increasing poverty (32). The UK COVID-19 Mental Health and Wellbeing Study has shown that mental ill-health is disproportionately impacting both young people and those with poorer socioeconomic status (33,34). Evidence indicates that the cost-of-living crisis will push an extra 600,000 individuals into poverty, and deepen existing poverty (35). These predictions are concerning given our findings, as we are likely to see a greater number of children and adolescents experiencing worse mental health.

### Strengths and Limitations

Key strengths of our study include the MCS data source. Given its national representation of UK adolescents, the evidence it provides can inform UK policy. Our research question examined mental health outcomes at age 17, which represents a sensitive transition period into adulthood, and enabled assessment of the impact of ASE throughout most of adolescence. Furthermore, multiple markers of ASE were measured, which have rarely been measured together in existing literature, resulting in a more robust assessment of ASE.

Limitations of our study include possible attrition bias. From an initial 10,625 participants in wave seven, there was attrition of 52% and 51% for our primary and secondary outcome variables respectively. Although weighted variables in our analysis accounted for attrition, drop-outs and non-response bias may still have underestimated adolescent mental ill-health (44). Reverse causation of maternal-adolescent mental ill-health was reduced by measuring maternal mental ill-health at 9 months (thus preceding adolescent mental ill-health), however we do not know the poverty or mental health status of mothers before entering the MCS. Furthermore, all mediators were assessed at the same timepoints, therefore interacting and limiting the ability to identify sensitive timepoints or ordered effects.

Although the Baron and Kenny approach helps to conclude whether an association is mediated by variable(s), it cannot quantify direct, indirect or total effects (45). We used this method because it enabled us to answer our research question of whether mediation exists, rather than estimating the percentage of mediation.

The measurement tools used also have limitations. Age 17 is the upper age limit for the SDQ, with some studies limiting to age 16, which may reduce the validity of our outcome measure (22,46). Furthermore, self-report may cause social desirability bias, and evidence also suggests that this may vary according to ethnicity (18). However, the prevalence of mental ill-health in adolescents in this study was 16-17%, which roughly concurs with national estimates (47).

The unadjusted association between exposure and outcome was not significant in all versions of the analyses. However, SDQ in our main analysis, and both K6 and SDQ in weighted linear regression analysis, were significant. The association between maternal-adolescent mental ill-health is also well-established in existing literature (4,48–50). Linear may have differed from logistic analysis due to harsh cut-off scores and therefore reduced sensitivity of the data in logistic models (36). Furthermore, effect size and model fits are considered appropriate measures of the strength and clinical significance of associations (51).

### Policy and practice implications

Our findings provide evidence to improve maternal health services around the time of birth, to prevent a direct pathway from maternal complications during labour to adolescent mental ill-health. Furthermore, given the evidence that these mothers may have subsequent poorer mental health (40,41), pathways from maternal mental ill-health to adolescent mental-ill health may also be prevented. Our additional analysis showing child mental health and development as mediators of the maternal-adolescent mental ill-health association suggests that early intervention to improve child mental health and development is likely to reduce adolescent mental ill-health.

This study refutes existing policy which solely blames mothers (52), since there are direct pathways from poverty through the life-course to adolescent mental ill-health operating independently of maternal mental health. Our findings suggest that even if born into poverty, adolescent mental health can be improved by reducing time in poverty. Earlier intervention to alleviate poverty is likely to have the greatest effect at reducing maternal and adolescent mental ill-health directly.

In conclusion, although cumulative ASE was not shown to mediate the maternal-adolescent mental ill-health association, we found several pathways to poor adolescent mental health. Maternal mental health impacts adolescent mental health through child mental health. High ASE in the early years impacts both maternal and child mental health, and increasing time spent in poverty increases the likelihood of adolescent mental ill-health. Maternal complications during labour (which may be triggered by maternal mental ill-health) represent another pathway to adolescent mental ill-health. Therefore, policy should be targeted towards early maternal health support *in addition to* raising household income. Acting in the early years to reduce time spent in poverty is likely to be pivotal to prevent and improve *both* maternal and adolescent mental ill-health.

Further research should include group-based trajectory modelling to examine ASE in more detail, for example, evaluating ASE as an early-life mediator, or as an outcome (53,54). This may facilitate tailored public health policy targeting sensitive timepoints and tackling multiple aspects of ASE together (53,54). Furthermore, counterfactual mediation analysis could quantify the proportion mediated by child mental health and development (23,55).

## Supplementary Material

Supplementary material

## Figures and Tables

**Table 1:** Descriptive characteristics of adolescents at age 17 using primary outcome variable (Kessler 6) (n=5,089). Chi-squared hypothesis tests were performed for each characteristic.

	Adolescents with mental ill-health (n=817)		Adolescents without mentalill-health (n=4272)		p-value
	%	n	%	n	
**Maternal mental health**					0.114
Mental ill-health	13.3	109	11.3	484	
No mental ill-health	86.7	708	88.7	3788	
**Maternal age**					0.237
12-19	4.7	38	4.2	179	
20-29	42.5	347	39.1	1669	
30-39	50.4	412	54.0	2305	
40+	2.4	20	2.8	119	
**Maternal ethnicity**					0.286
White	93.3	762	92.1	3935	
Mixed	0.6	5	0.7	29	
Indian	0.9	7	2.2	92	
Pakistani and Bangladeshi	2.6	21	2.4	101	
Black/Black British	1.7	14	1.8	75	
Other ethnic groups	1.0	8	0.9	40	
**Maternal education**					0.007
NVQ level 4-5	38.5	321	44.5	1903	
NVQ level 2-3	45.8	382	42.3	1806	
NVQ level 1, overseas, none	15.7	131	13.2	563	
**Relative Poverty**					0.002
Above 60% median income	75.4	616	80.1	3424	
Below 60% median income	24.6	201	19.9	848	
**Maternal Employment**					0.062
Employed	57.2	467	60.7	2594	
Not employed	42.8	350	39.3	1678	
**Housing**					0.001
Own or part rent/mortgage	70.4	575	76.2	3254	
Rent, living with parents, live rent free or squatting	29.6	242	23.8	1018	
**School readiness**					1.000
School ready	91.6	748	91.6	3912	
Not school ready	8.4	69	8.4	360	
**Maternal complications during labour**					0.495
No complications	64.1	524	65.4	2796	
Complications	35.9	293	34.6	1476	

Abbreviations: NVQ = National Vocational Qualification

**Table 2:** Summary of results of the main, additional and sensitivity analyses

	Model 1		Model 2		Model 3	
Primary outcome: Kessler 6 score in adolescents age 17 (n = 5,089)						
	OR	95% CI	AOR	95% CI	AOR	95% CI
**Maternal mental ill-health**						
**Main analysis**						
Weighted logistic regression	1.23	0.94,1.60	1.15	0.88,1.50	1.11	0.85,1.45
**Additional analysis**						
	-	-	-	-	1.06	0.80,1.39
**Sensitivity analysis**						
	1.21	0.97,1.51	1.15	0.91,1.43	1.12	0.89,1.39
	**β**	**95% CI**	**β**	**95% CI**	**β**	**95% CI**
Weighted linear regression	1.06	0.57,1.55	0.92	0.42,1.42	0.85	0.35,1.35
**Secondary outcome: SDQ score in adolescents age 17 (n = 5,227)**						
	**OR**	**95% CI**	**AOR**	**95% CI**	**AOR**	**95% CI**
**Maternal mental ill-health**						
**Main analysis**						
Weighted logistic regression	1.38	1.07,1.78	1.24	0.95,1.61	1.19	0.91,1.54
**Additional analysis**	-	-	-	-	0.99	0.75,1.30
**Sensitivity analysis**	1.30	1.05,1.61	1.19	0.95,1.47	1.15	0.92,1.43
	**β**	**95% CI**	**β**	**95% CI**	**β**	**95% CI**
Weighted linear regression	1.20	0.65,1.76	0.89	0.34,1.45	0.79	0.24,1.34

Abbreviations: 95% CI = 95% confidence interval; AOR = adjusted odds ratio; β = beta regression coefficient; OR = odds ratio; SDQ = Strengths and Difficulties Questionnaire; ASE = Adverse Socioeconomic Exposure

Model 1 = unadjusted analysis Model 2 = adjusted with confounders Model 3 = adjusted with confounders and cumulative ASE mediators Additional analysis = addition of child mental health and development mediators Sensitivity analysis = unweighted logistic regression

**Table 3:** Weighted logistic regression analysis

	Model 1: Unadjusted		Model 2: Adjusted with confounders		Model 3: Adjusted with confounders & cumulative ASE mediators	
Primary outcome: Kessler 6 score in adolescents age 17 (n = 5,089)						
	OR	95% CI	AOR	95% CI	AOR	95% CI
**Main exposure**						
Maternal mental ill-health	1.23	0.94,1.60	1.15	0.88,1.50	1.11	0.85,1.45
**Confounders (9 months)**						
Maternal complications during labour	-	-	1.15	0.96,1.39	1.14	0.95,1.37
Maternal age	-	-	1.00	0.86,1.17	1.02	0.87,1.19
Maternal ethnicity	-	-	0.99	0.89,1.10	0.97	0.86,1.08
Maternal education	-	-	1.07	0.93,1.22	1.00	0.87,1.15
Poverty	-	-	1.29	0.99,1.69	1.06	0.79,1.42
Housing	-	-	1.23	0.96,1.57	1.05	0.75,1.49
Employment	-	-	0.99	0.82,1.20	0.91	0.73,1.14
**Cumulative ASE mediators (3 to 14 years)**						
Employment	-	-	-	-	1.03	0.94,1.13
Housing	-	-	-	-	1.01	0.93,1.10
Poverty	-	-	-	-	1.15	1.04,1.26
**Secondary outcome: SD Q score in adolescents age 17 (n = 5,227)**						
	**OR**	**95% CI**	**AOR**	**95% CI**	**AOR**	**95% CI**
**Main exposure**						
Maternal mental ill-health	1.38	1.07,1.78	1.24	0.95,1.61	1.19	0.91,1.54
**Confounders (9 months)**						
Maternal complications	-	-	1.21	1.01,1.45	1.21	1.01,1.45
during labour						
Maternal age	-	-	0.89	0.77,1.03	0.91	0.79,1.06
Maternal ethnicity	-	-	0.87	0.76,1.00	0.85	0.74,0.98
Maternal education	-	-	1.17	1.02,1.33	1.07	0.93,1.24
Poverty	-	-	1.28	0.99,1.65	1.02	0.77,1.35
Housing	-	-	1.45	1.15,1.83	1.12	0.82,1.53
Employment	-	-	1.06	0.88,1.28	1.01	0.80,1.24
**Cumulative ASE mediators (3 to 14 years)**						
Employment	-	-	-	-	1.02	0.93,1.11
Housing	-	-	-	-	1.05	0.98,1.13
Poverty	-	-	-	-	1.16	1.05,1.27

Abbreviations: 95% CI = 95% confidence interval; AOR = adjusted odds ratio; OR = odds ratio; SDQ = Strengths and Difficulties Questionnaire; ASE = Adverse Socioeconomic Exposure

## Box

*What is already known on this subject?*

Overall, there is evidence that maternal mental health partially mediates the association between ASE and adolescent mental health. However, no study found that the association was completely mediated by maternal mental health, implying that there is also a direct effect from ASE to adolescent mental health. It has been suggested that ASE is a *confounder* of maternal and adolescent mental ill-health, but its role as a *mediator* has not been adequately explored.

*What this study adds?*

Combining multiple, cumulative ASE mediators across time, we found that cumulative ASE does not have a large mediation role between maternal and adolescent mental ill-health. However, we found that child mental health and development are mediators of the maternal-adolescent mental ill-health association. Experiencing cumulative poverty and maternal complications during labour also increase the risk of adolescent mental ill-health.

*Policy implications*

Early years interventions targeting both maternal health and poverty will likely benefit maternal and adolescent mental health directly.
