# Supplementary material for: Does cumulative adverse socioeconomic exposure mediate the association of maternal mental ill-health at birth and adolescent mental ill-health at age 17? An analysis of the Millennium Cohort Study
Source: J Epidemiol Community Health. Author manuscript; Available in PMC 2025 Sep 16. (PMC7618127; doi:10.1136/jech-2022-220248)
Supplement: Supplementary material [file EMS208291-supplement-Supplementary_material.docx]

**Supplementary Material**

**S1. Variables included in analysis and the cut-off scores used in the logistic regression models**

| **Variable** | **MCS variable code** | **Coding for analysis** | **Justification of cut off scores** |
| --- | --- | --- | --- |
| **Maternal mental ill-health** | *amtire00, amdepr00, amworr00, amrage00, amscar00, amupse00, amkeyd00, amnerv00, amhera00* | **0** = score <4 on Malaise Inventory  **1** = score of ≥4 on Malaise Inventory | As detailed by UK data service; cut off score of 4 as marker of depression/anxiety, as only 9 of the 24 questions were asked in MCS (78). |
| **Adolescent mental ill-health** | *gdckessl* | **0** = score <13 on Kessler 6  **1** = score ≥13 on Kessler 6 | Cut off score ≥13 has a 0.92 total classification accuracy (83,85) |
|  | *gebdtot_c* | **0** = score 0-16 on SDQ  **1** = score 17-40 on SDQ | Based on 4 categories. High or very high (score 17-40) and slightly raised or close to average (score 0-16) (86) |
| **Cumulative SDQ** | *bdebdta0, cdebdta0, ddebdta0, edebdtaa1, febdtot1* | **0** = score 0-16 on proxy reported SDQ  **1** = score 17-40 on proxy reported SDQ | Based on 4 categories. High or very high (score 17-40) and slightly raised or close to average (score 0-16) (86) |
| **Poverty** | *adoedp00, bdoedp00, cdoedp00, doedp000, eoedp000, foedp000* | **0** = >60% median household income  **1** = <60% median household income | Cut off of 60% was derived from MCS as per OECD threshold (90) |
| **Highest educational qualification** | *amdnvq00* | **0** = NVQ level 4 and 5  **1** = NVQ level 2 and 3  **2** = NVQ level 1, overseas or no qualifications | Grouped into three levels of higher maternal education (NVQ levels 4 or 5) (91), and GCSE C and above or A-levels (NVQ 2 or 3) and GCSE D-G (NVQ level 1), none or overseas qualifications (92) |
| **Employment status** | *amdwrk00, bmdwrk00, cmdwrk00, dmdwrk00, edcwrk00, fdcwrk00* | **0** = employed  **1** = not employed | Binary cut-off according to whether main respondent is in work or not (88) |
| **Housing tenure** | *adroow00, bdroow00, cdroow00, ddroow00, edroow00, fdroow00* | **0** = own or private rent  **1** = social rental, lives with parents, rent free, squatting or other | Four categories asked in MCS, split into two in terms of quality and stability of housing to broadly represent stable versus unstable accommodation (88) |
| **School readiness** | *bdsrcs001* | **0** = Bracken school readiness score ≥85 (school ready)  **1** = Bracken school readiness score <85 (not school ready) | Based on Bracken’s “normative classification”: very delayed, delayed, average, advanced, very advanced; adapted to “school ready” (>85) or “not school ready” (<85) i.e. above or below average as per Camacho et al. (93) |
| **Maternal complications during labour** | *comp_totbin* | **0** = no complications  **1** = any complications | Binary cut-off according to whether there were any complications during labour or not (88) |
| *Abbreviations: MCS = Millennium Cohort Study, NVQ = National Vocational Qualification, OECD = Organisation for Economic Co-operation and Development, SDQ = Strengths and Difficulties Questionnaire* | | | |

# S2. Boxplot of deciles of maternal mental health score by the outcome variable, adolescent mental health at age 17


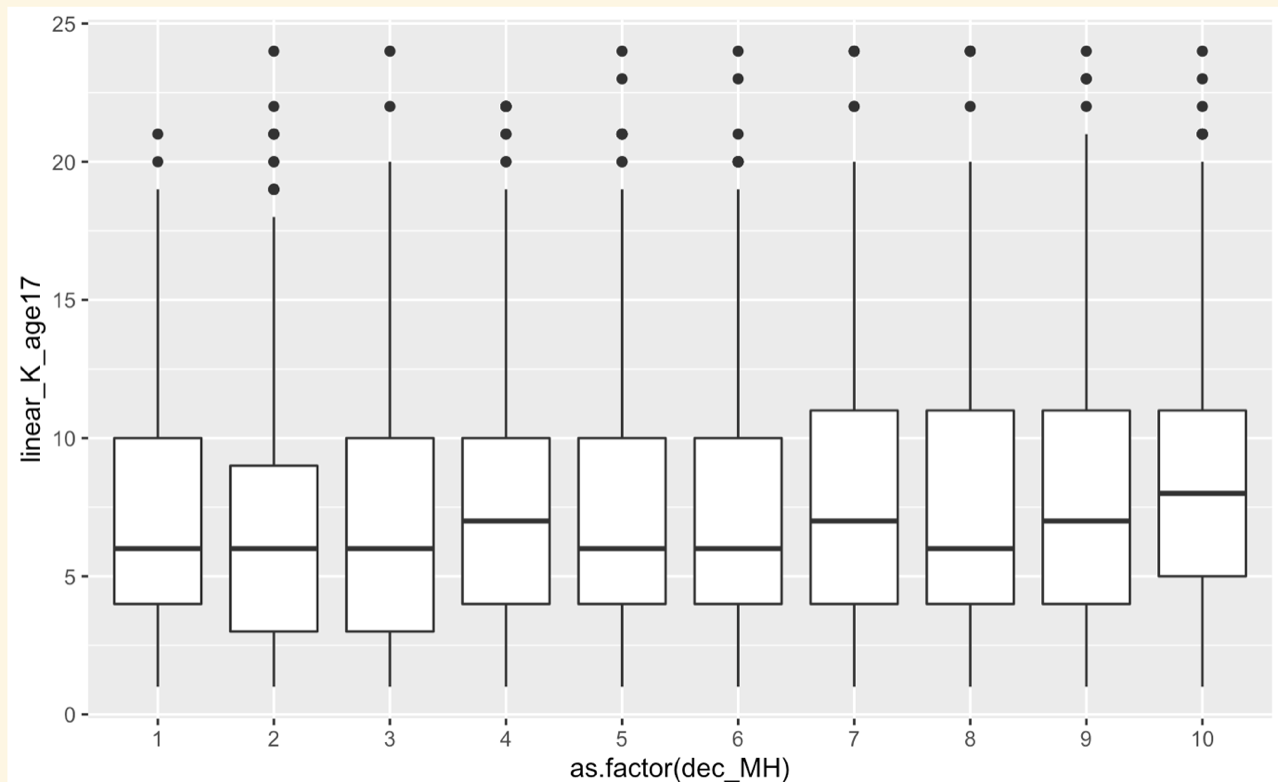


Continuous Kessler 6 score in adolescents at age 17

Deciles of maternal mental ill-health score around the time of child’s birth

## **S3. SDQ descriptive table (n=5227)**

|  | **Adolescents with mental ill-health at age 17**  **(n=871)** | | **Adolescents with no mental ill-health at age 17**  **(n=4356)** | | **p-value** |
| --- | --- | --- | --- | --- | --- |
|  | **%** | **n** | **%** | **n** |  |
| **Maternal mental ill-health** |  |  |  |  | 0.017 |
| Mental ill-health  (score >=13) | 14.0 | 122 | 11.1 | 484 |  |
| No mental ill-health  (score <13) | 86.0 | 749 | 88.9 | 3872 |  |
| **Maternal age** |  |  |  |  | <0.001 |
| 12-19 | 5.7 | 50 | 4.2 | 183 |  |
| 20-29 | 45.4 | 395 | 38.8 | 1688 |  |
| 30-39 | 46.2 | 402 | 54.4 | 2368 |  |
| 40+ | 2.8 | 24 | 2.7 | 117 |  |
| **Maternal ethnicity** |  |  |  |  | 0.007 |
| White | 94.5 | 823 | 91.7 | 3996 |  |
| Mixed | 1.0 | 9 | 0.6 | 25 |  |
| Indian | 0.7 | 6 | 2.1 | 93 |  |
| Pakistani and Bangladeshi | 1.7 | 15 | 2.7 | 117 |  |
| Black/Black British | 1.1 | 10 | 1.9 | 84 |  |
| Other ethnic groups | 0.9 | 8 | 0.9 | 41 |  |
| **Maternal education** |  |  |  |  | <0.001 |
| NVQ level 4-5 | 35.9 | 313 | 44.5 | 1937 |  |
| NVQ level 2-3 | 46.6 | 406 | 42.4 | 1846 |  |
| NVQ level 1, overseas, none | 17.5 | 152 | 13.2 | 573 |  |
| **Relative Poverty** |  |  |  |  | <0.001 |
| Above 60% median income | 72.2 | 629 | 80.3 | 3498 |  |
| Below 60% median  income | 27.8 | 242 | 19.7 | 858 |  |
| **Maternal employment** |  |  |  |  | <0.001 |
| Employed | 53.5 | 466 | 61.1 | 2661 |  |
| Not employed | 46.5 | 405 | 38.9 | 1695 |  |
| **Housing** |  |  |  |  | <0.001 |
| Own or part rent/mortgage | 65.7 | 572 | 76.7 | 3342 |  |
| Rent, living with parents, live rent free or squatting | 34.3 | 299 | 23.3 | 1014 |  |
| **School readiness** |  |  |  |  | 0.035 |
| School ready | 89.4 | 799 | 91.7 | 3995 |  |
| Not school ready | 10.6 | 92 | 8.3 | 361 |  |
| *Abbreviations: NVQ = National Vocational Qualification* | | | | | |

**S4. Weighted linear regression of maternal mental ill-health predicting mediator variables controlled for baseline confounders**

|  | **Maternal mental health as a predictor of mediators** | |
| --- | --- | --- |
|  | **β** | **95% CI** |
| **Cumulative ASE mediators** | |  |
| Poverty | 0.18 | 0.07,0.29 |
| Housing | 0.21 | 0.07,0.35 |
| Employment | 0.12 | 0.01,0.22 |
| *Abbreviations: 95% CI = 95% confidence interval; β = beta regression coefficient; ASE = adverse socioeconomic exposure* | | |
|  |  |  |

## **S5. Sensitivity Analysis – Unweighted Logistic Regression Models**

|  | **Model 1:**  **unadjusted** | | **Model 2:**  **adjusted with confounders** | | **Model 3:**  **adjusted with confounders & cumulative ASE mediators** | |
| --- | --- | --- | --- | --- | --- | --- |
| **Primary outcome: Kessler 6 score in adolescents age 17 (n = 5,089)** | | | | | | |
|  | **OR** | **95% CI** | **AOR** | **95% CI** | **AOR** | **95% CI** |
| **Main exposure** |  |  |  |  |  |  |
| Maternal mental ill-health | 1.21 | 0.96,1.51 | 1.15 | 0.91,1.43 | 1.12 | 0.89,1.39 |
| **Confounders (9 months)** | | |  |  |  |  |
| Maternal complications during labour | - | - | 1.09 | 0.93,1.28 | 1.09 | 0.93,1.28 |
| Maternal age | - | - | 0.99 | 0.87,1.12 | 1.01 | 0.89,1.14 |
| Maternal ethnicity | - | - | 0.95 | 0.86,1.03 | 0.92 | 0.84,1.01 |
| Maternal education | - | - | 1.08 | 0.96,1.21 | 1.01 | 0.89,1.14 |
| Poverty | - | - | 1.10 | 0.88,1.38 | 0.92 | 0.71,1.17 |
| Housing | - | - | 1.19 | 0.97,1.47 | 1.05 | 0.80,1.36 |
| Employment | - | - | 1.04 | 0.87,1.23 | 0.96 | 0.79,1.17 |
| **Cumulative ASE mediators (3 to 14 years)** | | | | | | |
| Employment | - | - | - | - | 1.02 | 0.94,1.10 |
| Housing | - | - | - | - | 1.00 | 0.94,1.07 |
| Poverty | - | - | - | - | 1.14 | 1.05,1.24 |
| **Secondary outcome: SDQ score in adolescents age 17 (n = 5,227)** | | | | | | |
|  | **OR** | **95% CI** | **AOR** | **95% CI** | **AOR** | **95% CI** |
| **Main exposure** |  |  |  |  |  |  |
| Maternal mental ill-health | 1.30 | 1.05,1.61 | 1.19 | 0.95,1.47 | 1.15 | 0.92,1.43 |
| **Confounders (9 months)** | | |  |  |  |  |
| Maternal complications during labour | - | - | 1.12 | 0.96,1.31 | 1.13 | 0.97,1.32 |
| Maternal age | - | - | 0.93 | 0.82,1.05 | 0.95 | 0.84,1.08 |
| Maternal ethnicity | - | - | 0.84 | 0.76,0.93 | 0.83 | 0.74,0.91 |
| Maternal education | - | - | 1.12 | 1.00-1.26 | 1.03 | 0.92,1.17 |
| Poverty | - | - | 1.12 | 0.90,1.40 | 0.91 | 0.72,1.16 |
| Housing | - | - | 1.43 | 1.17,1.75 | 1.14 | 0.88,1.47 |
| Employment | - | - | 1.14 | 0.96,1.34 | 1.09 | 0.90,1.31 |
| **Cumulative ASE mediators (3 to 14 years)** | | | | | | |
| Employment | - | - | - | - | 0.99 | 0.92,1.07 |
| Housing | - | - | - | - | 1.04 | 0.98,1.11 |
| Poverty | - | - | - | - | 1.15 | 1.07,1.24 |
| *Abbreviations: 95% CI = 95% confidence interval; AOR = adjusted odds ratio; OR = odds ratio; SDQ = Strengths and Difficulties Questionnaire; ASE = adverse socioeconomic exposure* | | | | | | |

**S6. Additional analysis - Logistic regression analyses with child health & development mediator variables compared to Model 2**

|  | **Model 2: adjusted with confounders** | | **Model 3 + child mental health and development mediators** | |
| --- | --- | --- | --- | --- |
| **Primary outcome: Kessler 6 score in adolescents age 17 (n = 5,089)** | | | | |
|  | **AOR** | **95% CI** | **AOR** | **95% CI** |
| **Main exposure** |  |  |  |  |
| Maternal mental ill-health | 1.15 | 0.88,1.50 | 1.05 | 0.80,1.39 |
| **Confounders (9 months)** | | |  |  |
| Maternal complications during labour | 1.15 | 0.96,1.39 | 1.13 | 0.94,1.36 |
| Maternal age | 1.00 | 0.86,1.17 | 1.03 | 0.88,1.20 |
| Maternal ethnicity | 0.99 | 0.89,1.10 | 0.97 | 0.87,1.09 |
| Maternal education | 1.07 | 0.93,1.22 | 1.00 | 0.86,1.15 |
| Poverty | 1.29 | 0.99,1.69 | 1.06 | 0.79,1.42 |
| Housing | 1.23 | 0.96,1.57 | 1.05 | 0.74,1.48 |
| Employment | 0.99 | 0.82,1.20 | 0.92 | 0.73,1.15 |
| **Cumulative mediators (3 to 14 years)** | | | | |
| Employment | - | - | 1.03 | 0.93,1.13 |
| Housing | - | - | 1.01 | 0.93,1.10 |
| Poverty | - | - | 1.14 | 1.04,1.26 |
| School readiness (age 3) | - | - | 0.79 | 0.56,1.12 |
| SDQ | - | - | 1.13 | 1.02,1.25 |
| **Secondary outcome: SDQ score in adolescents age 17 (n = 5,227)** | | | | |
|  | **AOR** | **95% CI** | **AOR** | **95% CI** |
| **Main exposure** |  |  |  |  |
| Maternal mental ill-health | 1.24 | 0.95,1.61 | 0.99 | 0.75,1.30 |
| **Confounders (9 months)** | |  |  |  |
| Maternal complications during labour | 1.21 | 1.01,1.45 | 1.17 | 0.98,1.41 |
| Maternal age | 0.89 | 0.77,1.03 | 0.94 | 0.81,1.09 |
| Maternal ethnicity | 0.87 | 0.76,1.00 | 0.85 | 0.74,0.99 |
| Maternal education | 1.17 | 1.02,1.33 | 1.04 | 0.90,1.21 |
| Poverty | 1.28 | 0.99,1.65 | 1.00 | 0.75,1.33 |
| Housing | 1.45 | 1.15,1.83 | 1.08 | 0.79,1.49 |
| Employment | 1.06 | 0.88,1.28 | 1.01 | 0.81,1.26 |
| **Cumulative mediators (3 to 14 years)** | | | | |
| Employment | **-** | - | 1.01 | 0.92,1.11 |
| Housing | - | - | 1.05 | 0.97,1.13 |
| Poverty | - | - | 1.12 | 1.02,1.24 |
| School readiness (age 3) | - | - | 0.96 | 0.70,1.33 |
| SDQ | - | - | 1.45 | 1.32,1.60 |
| *Abbreviations: 95% CI = 95% confidence interval; AOR = adjusted odds ratio; SDQ = Strengths and Difficulties Questionnaire* | | | | |

##

## **S7. Sensitivity Analysis - Weighted Linear Regression Models**

|  | **Linear Model 1:**  **Unadjusted** | | **Linear Model 2:**  **Adjusted with confounders** | | **Linear Model 3:**  **Adjusted with confounders & cumulative ASE mediators** | |
| --- | --- | --- | --- | --- | --- | --- |
| **Primary outcome: Kessler 6 score in adolescents age 17 (n = 5,089)** | | | | | | |
|  | **β** | **95% CI** | **β** | **95% CI** | **β** | **95% CI** |
| **Main exposure** |  |  |  |  |  |  |
| Maternal mental ill-health | 1.06 | 0.57,1.55 | 0.92 | 0.42,1.42 | 0.85 | 0.35,1.35 |
| **Confounders (9 months)** | | | |  |  |  |
| Maternal complications during labour | - | - | 0.14 | -0.18,0.46 | 0.14 | -0.18,0.46 |
| Maternal age | - | - | -0.17 | -0.44,0.11 | -0.12 | -0.40,0.15 |
| Maternal ethnicity | - | - | -0.10 | -0.28,0.07 | -0.13 | -0.31,0.04 |
| Maternal education | - | - | 0.07 | -0.17,0.31 | -0.06 | -0.30,0.19 |
| Poverty | - | - | 0.46 | -0.06,0.99 | 0.07 | -0.48,0.63 |
| Housing | - | - | 0.46 | -0.00,0.91 | -0.04 | -0.64,0.56 |
| Employment | - | - | 0.06 | -0.27,0.38 | -0.05 | -0.42,0.33 |
| **Cumulative ASE mediators (3 to 14 years)** | | | | | | |
| Employment | - | - | - | - | 0.03 | -0.13,0.19 |
| Housing | - | - | - | - | 0.12 | -0.03,0.26 |
| Poverty | - | - | - | - | 0.24 | 0.05,0.42 |
| **Secondary outcome: SDQ score in adolescents age 17 (n = 5,227)** | | | | | | |
|  | **β** | **95% CI** | **β** | **95% CI** | **β** | **95% CI** |
| **Main exposure** |  |  |  |  |  |  |
| Maternal mental ill-health | 1.20 | 0.65,1.76 | 0.89 | 0.34,1.45 | 0.79 | 0.24,1.34 |
| **Confounders (9 months)** | | |  |  |  |  |
| Maternal complications during labour | - | - | 0.24 | -0.12,0.60 | 0.24 | -0.12,0.60 |
| Maternal age | - | - | -0.34 | -0.64,-0.03 | -0.27 | -0.57,0.04 |
| Maternal ethnicity | - | - | -0.41 | -0.61,-0.20 | -0.45 | -0.66,-0.24 |
| Maternal education | - | - | 0.46 | 0.19,0.74 | 0.27 | -0.02,0.55 |
| Poverty | - | - | 0.72 | 0.15,1.29 | 0.09 | -0.52,0.70 |
| Housing | - | - | 1.10 | 0.59,1.60 | 0.34 | -0.31,0.99 |
| Employment | - | - | -0.10 | -0.48,0.28 | -0.16 | -0.59,0.27 |
| **Cumulative ASE mediators (3 to 14 years)** | | | | | | |
| Employment | - | - | - | - | -0.04 | -0.22,0.15 |
| Housing | - | - | - | - | 0.17 | 0.01,0.33 |
| Poverty | - | - | - | - | 0.40 | 0.20,0.61 |
| *Abbreviations: 95% CI = 95% confidence interval; β = beta regression coefficient; SDQ = Strengths and Difficulties Questionnaire; ASE = adverse socioeconomic exposure* | | | | | | |
